# Rheopathologic Consequence of *Plasmodium vivax* Rosette Formation

**DOI:** 10.1371/journal.pntd.0004912

**Published:** 2016-08-10

**Authors:** Rou Zhang, Wenn-Chyau Lee, Yee-Ling Lau, Letusa Albrecht, Stefanie C. P. Lopes, Fabio T. M. Costa, Rossarin Suwanarusk, Francois Nosten, Brian M. Cooke, Laurent Rénia, Bruce Russell

**Affiliations:** 1 Department of Microbiology and Immunology, Yong Loo Lin School of Medicine, National University of Singapore, National University Health System, Singapore; 2 Singapore Immunology Network (SIgN), Agency for Science, Technology and Research (A*STAR), Singapore; 3 Department of Parasitology, Faculty of Medicine, University of Malaya, Kuala Lumpur, Malaysia; 4 Laboratory of Tropical Diseases, Instituto de Biologia, Universidade Estadual de Campinas (UNICAMP), Campinas-SP, Brazil; 5 Shoklo Malaria Research Unit, Mahidol-Oxford tropical Medicine Research Unit, Faculty of Tropical Medicine, Mahidol University, MaeSot, Thailand; 6 Centre for Tropical Medicine, Nuffield Department of Medicine, University of Oxford, Oxford, United Kingdom; 7 Programs in Infection and Immunity and Cardiovascular Disease, Monash Biomedicine Discovery Institute and Department of Microbiology, Monash University, Victoria, Australia; 8 Department of Microbiology and Immunology, University of Otago, Dunedin, New Zealand; New York University School of Medicine, UNITED STATES

## Abstract

Malaria parasites dramatically alter the rheological properties of infected red blood cells. In the case of *Plasmodium vivax*, the parasite rapidly decreases the shear elastic modulus of the invaded RBC, enabling it to avoid splenic clearance. This study highlights correlation between rosette formation and altered membrane deformability of *P*. *vivax*-infected erythrocytes, where the rosette-forming infected erythrocytes are significantly more rigid than their non-rosetting counterparts. The adhesion of normocytes to the *Pv*IRBC is strong (mean binding force of 440pN) resulting in stable rosette formation even under high physiological shear flow stress. Rosetting may contribute to the sequestration of *Pv*IRBC schizonts in the host microvasculature or spleen.

## Author Summary

While *Plasmodium vivax* is generally not as virulent as *P*. *falciparum*; severe manifestations of vivax malaria do occur. While little is known about the mechanisms underlying the pathobiology of *P*. *vivax*, most agree its ability to increase the deformability of stiff host reticulocytes is key adaptation to avoid splenic clearance. We show that *P*. *vivax*-infected red blood cells (*Pv*IRBCs) rosette irreversibly with normocytes and are significantly more stiff than non-rosetting *Pv*IRBCs. We discuss how these stiff *Pv*IRBC rosettes are removed from the peripheral circulation and its rheopathological consequences.

## Introduction

*Plasmodium* spp. derived changes to the rheology of infected red blood cells (IRBCs) play a central role in the pathogenesis of human malaria. Malaria parasite remodelling of IRBCs dramatically alter their deformability and cytoadhesive properties [1]. Interestingly, for all four non-zoonotic causes of human malaria (*P*. *falciparum*, *P*. *vivax*, *P*. *ovale* and *P*. *malariae*) IRBCs cytoadhere to uninfected RBCs forming distinctive ‘rosettes’ [2–4]. While the precise role of rosetting in malaria pathogenesis remains contentious, many believe that this adaptation may play important roles in the survival of parasites within the circulation [5]. Rheological studies on *P*. *falciparum* rosettes show them to be stable and the binding force between the IRBC and the uninfected RBCs tends to be very strong (>300pn) [6]. Indeed, most studies on rosetting have focused on *P*. *falciparum*, leading to the discovery of rosetting ligands such as PfEMP1 [7], STEVOR [8], and RIFINs [9]. Although rosette formation has been reported to be a common phenomenon in *P*. *vivax* [2, 10, 11], the rosetting ligand of this species has yet to be discovered. Despite recent evidence showing cytoadhesive potential for *P*. *vivax*-infected RBCs [12], most consider this species to be much less adhesive than *P*. *falciparum*, as it lacks any orthologue to the *Pf*EMP1 protein (the key cytoadhesive ligand in *P*. *falciparum*) and the knobby IRBC ultrastructure (which concentrate and display *Pf*EMP-1) that facilitate binding of IRBCs to the vascular endothelium under physiological shear flow [13]. Therefore, although *P*. *vivax* rosettes are relatively commonly observed, it is not known whether they are stable structures or ephemeral *ex-vivo* formations that break apart in the haemodynamic environment of the circulation *in vivo*. The objective of this study was to examine the rheological consequences of rosetting on *Pv*IRBCs and specifically quantify the binding strength of normocytes to *Pv*IRBCs.

## Methods

### Ethics statement

Blood samples of vivax malaria patients from the Northwestern Thailand were collected under the following ethical guidelines and approved protocols: OXTREC 027–025 (University of Oxford, Centre for Clinical Vaccinology and Tropical Medicine, UK) and MUTM 2008–215 from the Ethics Committee of Faculty of Tropical Medicine, Mahidol University, Thailand. Experiments were conducted in Singapore Immunology Network (SIgN) and National University of Singapore (NUS), Singapore. All adult subjects provided informed written consent, and a parent or guardian of any child participant provided informed written consent on their behalf. Ten clinical samples were collected from malaria patients of SMRU clinics in Northwestern Thailand using BD Vacutainer with lithium heparin anticoagulant. Thick and thin blood smears were prepared for each sample to determine the species of malaria parasite, the parasitemia, and the predominating developmental stage of the parasite. White blood cells were depleted with cellulose (Sigma-Aldrich) packed columns. Blood samples containing predominantly ring-stage parasites (≥ 70%) were cryopreserved with Glycerolyte 57 (Fenwal). For experiments, cryopreserved isolates were thawed and the parasites matured *in vitro* [14]. When the parasite population reached late erythrocytic stages (late trophozoite and schizont), 50 μl of the culture suspension was taken for rosetting assay using a wet mount method as described elsewhere[11]. Rosetting rate (percentage of rosette-forming IRBCs) was determined by examining the number of of rosettes per 200 IRBCs observed. Subsequently, 1 μl packed RBCs were suspended in 1 ml of 1X PBS supplemented with 1% BSA for micropipette aspiration and microfluidic assays.

Micropipette aspiration was modified from Hochmuth et al [15]. Briefly, aspiration was performed at 32°C to 37°Cand observed using an oil immersion objective (1000 x magnification) with an Olympus research inverted microscope IX73. Borosilicate glass micropipettes (diameter 1.5±0.2 μm) were used to hold or aspirate RBCs. Rosetting and non-rosetting IRBCs were individually selected for measurements. Individual RBCs were aspirated at a pressure drop rate of 0.5 Pa/s for 100s. The corresponding cell membrane deformation was recorded using the Dual CCD Digital Camera DP80 (Olympus) at an image taking rate of one frame/s. Images were processed by cellSens Dimension (Olympus). Hemispherical cap model was used to calculate the membrane shear elastic modulus, as a quantitative surrogate measure of the rigidity of RBC membrane skeleton [15].

To quantify the binding force between RBCs and an IRBC in a *P*. *vivax* rosette, a double pipette aspiration method was used as described previously [6]. A rosette was held by a micropipette (diameter = 2.0±0.2μm). A second micropipette was used to aspirate the uninfected RBCs of the rosette at a gradually increased aspirating pressure. The force (F) to detach an RBC from an IRBC was calculated as F = πr^2^ × P; where r is the inner diameter of the second micropipette, and P is the pressure required to detach two cells. The aspiration pressure was measured by a pressure transducer (P61 model, Validyne Engineering) and recorded by USB-COM Data logger (Validyne Engineering). The process was recorded using a Dual CCD Digital Camera DP80 (Olympus) at one frame/s. Recorded images were analyzed with cellSens Dimension (Olympus).

To characterize the ability of *Pv*IRBCs to move through narrow channels, polydimethylsiloxane (PDMS) microfluidic chips with 4μm slits were used. To avoid RBCs from interacting with (or adhering to) the walls of the microfluidic chip, channels were pre-filled and incubated with 1X PBS supplemented with 1% BSA for one hour prior to the experiment being performed. Subsequently, 1μl of RBC suspension was injected into the microfluidic channel. Cells were forced through the channel at a constant pressure gradient of 0.1 Pa/μm. Numbers of RBCs that blocked at the openings of the microfluidic channels in each experiment were recorded. Videos of the microfluidic assay were recorded using a Dual CCD Digital Camera DP80 (Olympus). Data were subsequently analyzed using the cellSens Dimensions software (Olympus). GraphPad Prism 5 was used for statistical analysis of all experimental data. The one-way ANOVA test was used to compare differences between different experimental groups.

## Results

**Fig 1:**
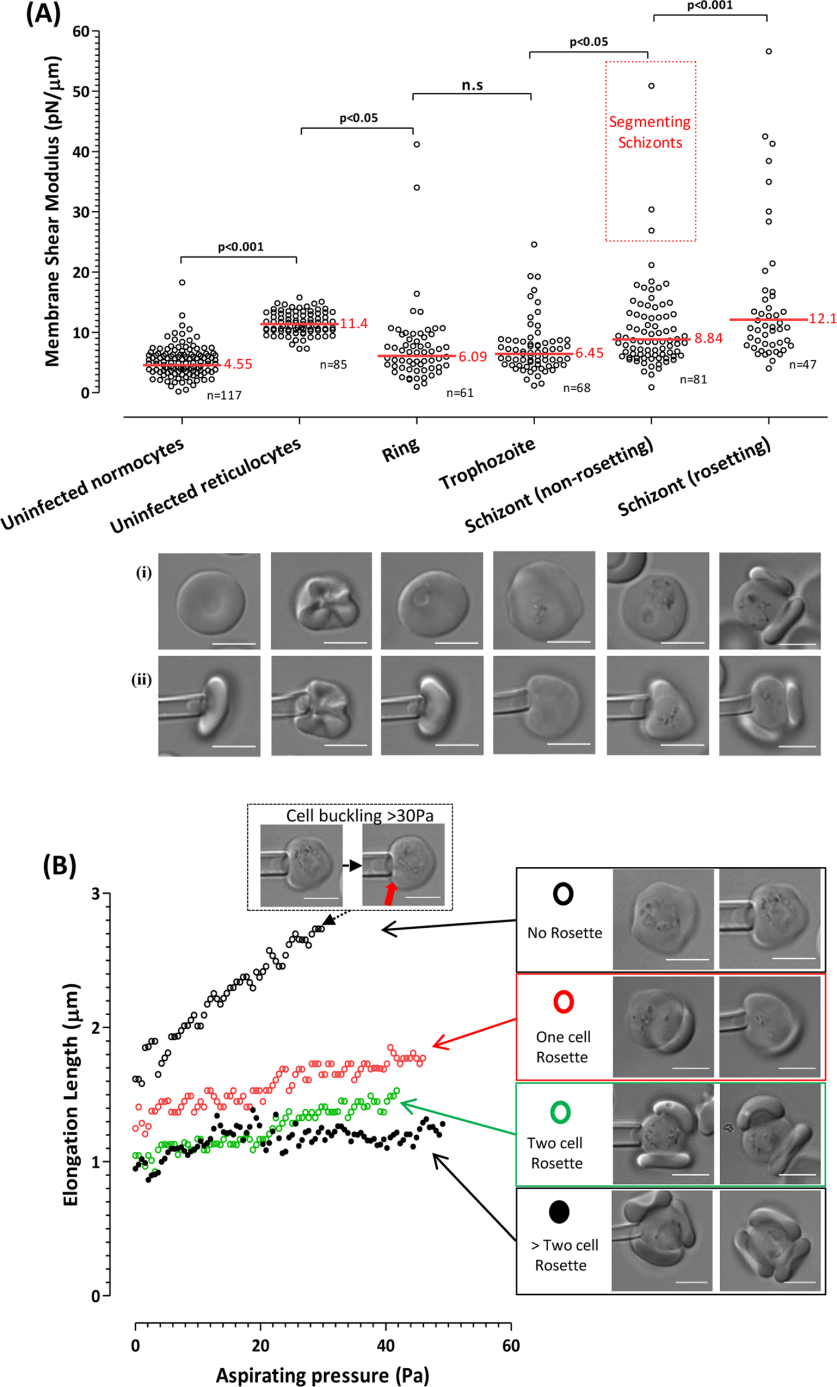
(A) The effect of *P*. *vivax* invasion, development and rosetting on the deformability of the infected reticulocyte membrane (normocytes are shown as a comparator). Plot showing membrane shear moduli (SM) (a higher SM indicates a reduced membrane deformability) of different cell types and stages of *P*. *vivax* erythrocytic development, with geometric mean (overall of 10 isolates) SM of each group indicated by a red line (each dot represents an individual cell measurement the total ‘n = x’). Pictures of respective cell types before (i) and during (ii) membrane shear modulus measurement by micropipette aspiration are shown under the graph. Mean (Geometric) shear moduli was compared using ANOVA (Bonferroni correction) and multiple comparison test (tukey). Uninfected normocytes were significantly more deformable than uninfected reticulocytes (P< 0.001). However both ring and trophozoite *P*. *vivax* stages become progressively more deformable (P< 0.05) until schizont stage (the very mature schizonts ‘segmenters’ were especially rigid). When normocytes adhered (rosette) with schizonts the infected cell membrane became significantly more rigid than non-rosetting schizonts (P< 0.001). (B) The number of normocytes involved in the rosette had no significant effect on the mean deformability kinetics (aspirated length versus the suction pressure) of the IRBCs. Pictures of cells before (i) and during (ii) measurement are shown under the graph. Measurements were done with increasing aspirating pressure until cells became structurally unstable under that pressure point (buckling effect), as shown by pictures (inset).

In keeping with previous report [11], cryopreserved *P*. *vivax* isolates showed rosetting, albeit with lower frequency than the fresh isolates. The rosettes found in these cryopreserved isolates were generally small. A mode of three uninfected normocytes were involved in rosettes (Fig 1). Similar to the previous study [11], rosetting in this study was only observed with RBCs infected with the late erythrocytic stages (predominantly schizonts).

Membrane shear elastic modulus measurements were used to quantify IRBC membrane deformability (Fig 1A). Uninfected reticulocytes showed significantly higher membrane shear moduli than uninfected normocytes (11.40±1.85 pN/μm vs. 4.55±2.58 pN/μm; P < 0.001). Interestingly, the membrane shear elastic moduli of *P*. *vivax* ring-infected reticulocytes were reduced to values similar to uninfected normocytes (6.09±6.45 pN/μm). The membrane shear elastic moduli of IRBCs remained virtually unchanged at the trophozoite stage (6.45±4.31 pN/μm). The membrane shear elastic modulus of non-rosetting schizonts were significantly higher than measurements recorded by trophozoites (8.84±6.88 pN/μm; P < 0.05). Measurements performed on rosetting schizonts (12.1±11.36 pN/μm) were significantly higher than those of non-rosetting schizonts (P < 0.01).

All RBCs showed an increased elongation length (i.e. increased deformability) with increasing aspiration pressure (Fig 1B). The attachment of a single uninfected RBC caused a significant reduction in deformability of the IRBC (P < 0.05). However, a Spearman’s rank correlation analysis showed that the attachment of additional RBCs did not result in further decreases to IRBC deformability, regardless of the size of the rosettes formed (Fig 1B).

**Fig 2:**
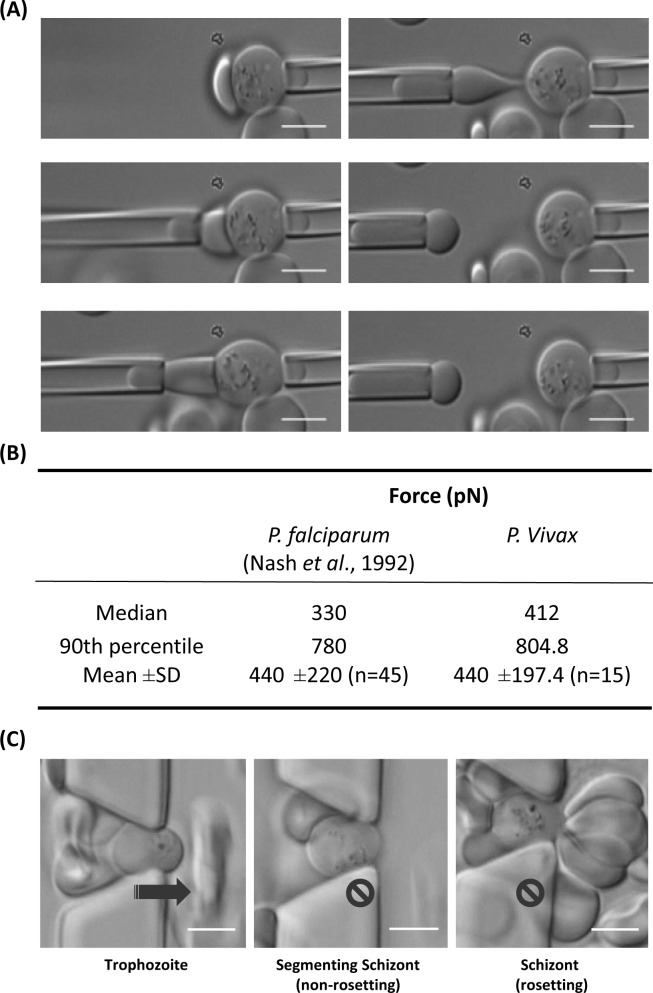
(A) Binding affinity of the rosetting complex using dual micropipette aspiration technique (B) Comparison of binding forces recorded from *P*. *vivax* rosettes (from this study) and *P*. *falciparum* rosettes (Nash et al 1992). (C) Examples of IRBCs capable of moving through 4 um microfluidic channel openings (Trophozoites (first image)) at 0.1 Pa and those that are trapped mature schizonts.

From dual micropipette aspiration assays (Fig 2A) (S1 Video), the shear force to separate uninfected RBCs from a rosetting complex was 440±197.4pN, which was similar to that reported previously for *P*. *falciparum* [6] (Fig 2B). In microfluidic experiments (Fig 2C), RBCs infected with either *P*. *vivax* ring, trophozoite or schizonts (early schizont and mature segmenting schizont) stages (Three clinical isolates in total were used) were injected into microfluidic channels as previously shown (S2–S5 Videos) [16]. The only cells observed blocking the microfluidic restrictions were rosetting and very mature segmenting schizonts. Rosettes blocking the microfluidic restrictions did not lose cells under pulsed shear flow pressure up to of 1.0 Pa/μm.

To better determine if the act of rosetting directly causes changes to the IRBC shear modulus (as opposed to IRBCs with a higher shear modulus are more likely to form rosettes) we measured the shear modulus of rosetting IRBCs, then using the dual micropipette we carefully peeled off the uninfected normocytes and repeated the measurement on the denuded IRBC. As the rosetting cells strongly bind to the IRBC, the separation process usually resulted the destruction of the IRBC. We were able to conduct 5 successful paired rosette separations, showing a significant reduction in the mean geometric shear modulus of the IRBC from 13.3pN (Rosetting) to 9.5pN (Non-Rosetting) (P<0.05, t = 2.8, df = 4(Paired t-test)).

## Discussion

*Plasmodium vivax*, the most globally-widespread cause of human malaria, has a specific tropism for the rigid CD71+ve reticulocytes generally found in the bone marrow [14, 17]. Within six hours post invasion, *P*. *vivax* remodels the IRBC membrane and cytoskeleton, causing it to become as deformable as an uninfected normocyte [14, 18]. In contrast to *P*. *falciparum*, RBCs infected with trophozoite and early schizont stages of *P*. *vivax* retain a relatively low shear modulus (compared to reticulocytes and *P*. *falciparum* IRBCs), and are able to deform and pass through micro-capillaries and 2μm sinusoidal slits [16]. It is thought that *P*. *vivax* increases the deformability of the host cell to avoid splenic clearance [18].

Our results show that rosetting with at least one uninfected RBC is closely associated with a a significant increase in the rigidity of the *P*. *vivax* IRBCs. While it is difficult to demonstrate direct causality, we were able to demonstrate that the removal of rosetting RBCs, restores the deformability of the IRBC to the levels usually seen in non rosetting IRBCs.

It is Important to understand that these rosettes are stable even under shear stress, and on encountering microfluidic constrictions they not only block the restriction, but also retain their full complement of attached uninfected red cells. The only other *P*. *vivax* IRBCs that tend to block the microfluidic restrictions are very mature schizonts. Traditionally these very late stage schizonts are referred to as ‘segmenters’, because the merozoites are fully mature and clearly defined within the schizont complex. In *P*. *falciparum*, late stage asexual parasites become rigid due to a range of proteins such as RESA, KHARP, MESA, PfEMP3 and STEVOR interacting with the IRBC cytoskeleton and membrane[1, 19–23]. In *P*. *vivax* we do not understand the molecular basis driving the switch from a relatively deformable early schizonts, to a rigid segmenter. However, as this change occurs an hour or so before schizonts rupture; we speculate the rigidity in *P*. *vivax* segmenters is due to osmotic deregulation (as opposed to the incorporation of crosslinking proteins into the cytoskeleton) as the IRBC membrane degenerates prior to merozoite release. In any case, our study clearly demonstrates that segmenting schizonts and rosetting are the only events responsible for significant rigidity of the *P*. *vivax* IRBCs.

Recent studies in Brazilian individuals infected with *P*. *vivax* reveal a disparate and unexpected disappearance of schizonts from the circulation [24]. Although this may be partially due to cytoadherence to endothelial receptors expressed on the surface of the vascular endothelium [12], we suggest that the increased rigidity of segmenters and rosetting IRBCs is a major factor behind the paucity of *P*. *vivax* schizonts in the circulation. The ligands responsible for *P*. *vivax* rosetting remain unknown. The *vir* proteins of *P*. *vivax* have been associated with endothelial cytoadhesion [12].

While we still expect to see spontaneous rosette formation occurring in the circulation, our study suggests that a large proportion *P*. *vivax* rosettes will be sequestered. Although the incidence and rate of *P*. *vivax* rosetting is high, we are still unsure how this phenomenon contributes to the pathology of vivax malaria[25]. It is important to understand that while rosetting has been observed in most forms of human malaria[2–4, 26], we only have a a clear understanding of this process in *P*. *falciparum*. Future studies should strive to understand the pathobiological process behind non-falciparum and possible develop therapeutics that disrupt their formation[27, 28].

## Supporting Information

S1 Video Dual micropipette aspiration technique was applied to detach the uninfected erythrocyte adhered to infected erythrocytes. Force required to dissociate the rosette was recorded.
(AVI)

S2 Video Microfluidic assay on one recruited *P*. *vivax* infected sample. The video showed the unblocked flow condition, where cells moved through the channel openings rapidly and identity of the cells (infected and uninfected) cannot be differentiated clearly from the video.
(AVI)

S3 Video Microfluidic assay video showing a non-rosette forming trophozoite-infected erythrocyte wiggling through the channel opening with slight impediment. (AVI)

S4 Video Microfluidic assay video showing a non-rosette forming segmenting schizont-infected erythrocyte being blocked at the channel opening. Other cells were seen passing through the channel opening.
(AVI)

S5 Video Microfluidic assay video showing a rosette forming schizont infected erythrocyte being blocked at the channel opening. Participating uninfected erythrocytes of the rosette did not detach from the blockade to move freely, showing the stability of the rosetting complex.
(AVI)
